# 0D/2D Mixed Dimensional Lead-Free Caesium Bismuth Iodide Perovskite for Solar Cell Application

**DOI:** 10.3390/ma15062180

**Published:** 2022-03-16

**Authors:** Salma Maneno Masawa, Jihong Li, Chenxu Zhao, Xiaolong Liu, Jianxi Yao

**Affiliations:** 1 Beijing Key Laboratory of Energy Safety and Clean Utilization, North China Electric Power University, Beijing 102206, China; salma.masawa@udom.ac.tz (S.M.M.); lijihong2413@gmail.com (J.L.); zhaochenxuphd@gmail.com (C.Z.); xl.liu@ncepu.edu.cn (X.L.); 2 Department of Petroleum and Energy Engineering, College of Earth Sciences and Engineering, The University of Dodoma, Dodoma P.O. Box 259, Tanzania; 3 State Key Laboratory of Alternate Electrical Power System with Renewable Energy Sources, North China Electric Power University, Beijing 102206, China

**Keywords:** perovskite solar cells, lead-free, solvent vapour annealing, caesium bismuth iodide

## Abstract

Bismuth-based perovskites are potentially a promising alternative for lead-free perovskites. During bond formation, however, trivalent ions on ${\mathrm{Cs}}_{3}{\mathrm{Bi}}_{2}I_{9}\;$with CsI/BiI_3_ ratio of 1.5/1 form 0D-neutral charged compounds with higher bandgap (>2.0 eV) and poor absorption capacity. Mixed 0/2-dimensional structures are potentially suitable substitutes due to their low bandgap. So far, the reported CsI/BiI_3_ ratios for 0D/2D structures are 1:1, 1:2 and 1:3. Herein, a new ratio of 1/1.5 is reported. Caesium bismuth iodide at a ratio of CsI/BiI_3_ of 1/1.5 was synthesised using a one-step processing method with/without solvent vapour annealing. During solvent annealing, a 1/4 (*v*/*v*) mixture of DMF/methanol was used as a solvent. The crystal structure formed at a ratio of 1/1.5 is more similar to 1.5/1 than to 1/3. The XRD pattern revealed additional characteristics peaks at 009, 012, 209 and 300, indicating the growth of another phase. The formed heterogeneous mixed 0D/2D structure has an extended light absorption capacity greater than 720 nm. Solvent vapour annealing improved film morphology by enhancing grain size and packing density. When cells with and without solvent vapour annealing are compared, the power conversion efficiency of caesium bismuth iodide increases from 0.26% without solvent annealing to 0.98% with solvent vapour annealing. This study establishes a new route for future research on crystal configuration, nomenclature, film and morphology, quality tailoring and applications toward the goal of lead-free perovskite solar cells.

## 1. Introduction

Organic-inorganic lead halide perovskite solar cells have paved the way for low-cost thin-film solar cells. Tremendous improvement in power conversion efficiency and easiness of the fabrication method have triggered great attention from 2009 to 2021, efficiency has increased from 3.8% to 25.7% [1,2,3,4]. However, lead is known to be toxic to the ecosystem and human health, potentially affecting its propagation [2,5,6,7,8,9]. Moreover, poor stability and hysteresis effect hinder progress towards commercialisation [9,10,11,12]. The best way to achieve commercialisation of thin-film low-cost perovskite solar cells is by considering all inorganic lead-free perovskites [13,14,15]. Miyasaka et al. [16] reported that the SQ efficiency of lead-free narrow bandgap solar absorbers can be higher (about 35%) than that of lead-based solar absorbers. The search for lead replacement revealed that the lead group-mates such as Sn and Ge could be suitable candidates [17,18,19,20,21]. However, these elements exhibit poor stability under ambient conditions as well as a potentially harmful resultant degradation product $\left({\mathrm{SnI}}_{2}\right)\;$ [22,23].

Bismuth-based perovskite materials seem to be a promising alternative for lead-free perovskites due to their suitable optoelectronic properties, high defect tolerance, less toxicity and good stability towards humidity, light and heat [2,24,25]. The effective ionic radius of bismuth (1.03° A) is equivalent to that of lead ions  (1.19° A). Further, it possesses analogous electronegativity and the same $6S^{2}$ lone pair on the valence shell as ${\mathrm{Pb}}^{2+}$ [26]. Additionally, due to $\;{\mathrm{BiX}}_{6}^{3-}\;$ octahedron structure, bismuth-based solar cells can generate perovskite structures with diverse dimensionality similar to that of lead [27].

Bismuth iodide perovskite solar cells materials that employ caesium as the A-site cation possesses high absorption coefficients and higher stability with uniform and dense morphology [28]. The first 0-D ${\mathrm{Cs}}_{3}{\mathrm{Bi}}_{2}I_{9}$ solar cell, with the ratio of $\left(\mathrm{CsI}:{\mathrm{BiI}}_{3}\right),\;\;\left(1.5:1\right)$ was fabricated by B. W. Park et al., 2015. Due to its dimensionality, the cell was found to have large indirect bandgap (2eV), but with poor quality of the films due to low rate of crystal formation and surface coverage, poor photocurrent density as well as poor interfacial contact between hole and electron transport materials [28]. The introduction of a new molar ratio of 1:3 led to the formation of a new compound ${\mathrm{CsBi}}_{3}I_{10}$ with reduced bandgap (1.77eV). The cell was also found to have extended visible light absorption spectrum at longer wavelength which exceeded that of$\;{\mathrm{Cs}}_{3}{\mathrm{Bi}}_{2}I_{9}$ by approximately 100 nm [29]. The effect of various hole transport materials was studied elsewhere [30,31,32,33]; it was found that the use of inorganic hole transport material or dopant-free hole materials can enhance the solar cell performance and stability.

Solvent vapour annealing (SVA), anti-solvent crystallisation (AC) or combined method (both AC and SVA) have also been employed to tailor the film morphology, with significant improvement [30,33,34,35]. Shin et al. [34] reported that using an anti-solvent during spinning on bismuth-based perovskites can effectively control the nucleation and growth rates, resulting in an increase in fill factor and thus power conversion efficiency. The comparison between the three most common types of solvent; toluene, isopropanol (IPA) and chlorobenzene was conducted by Ghosh et al. [30]. Toluene displayed higher power conversion efficiency compared to isopropanol and chlorobenzene. The use of mixed solvents such as chlorobenzene and isopropyl alcohol or ethyl ether and n-hexane has also been reported to slow down the perovskite film formation, facilitating the film with high nucleation density and crystal grains with good orientation and smooth surface. Zhu et al. [31] used 4-tert-butylpyridine (TBP) as an anti-solvent to enhance the film quality of CsBi_3_I_10_. SEM images showed that the TBP recrystallisation tends to enlarge the crystals of the formed film. The anti-solvent tends to remove the excess CsI ions and bismuth iodide, causing early nucleation and increase of grain size, consequently reducing the defect density of the cell [31].

Liang et al. [35] observed high phase purity and better crystallinity with dense morphology whenCsBi_3_I_10_ perovskite film was fabricated using a one-step processing method under solvent vapour annealing with a mixture of DMF/DMSO vapour. Khadka et al. [33] conducted a comparative study using solvent annealing, anti-solvent crystallisation or combined method on the optophysical and structural properties of caesium bismuth iodide perovskite. Uniform film with compact morphology was observed when chlorobenzene was used during anti-solvent crystallisation, followed by DMF solvent vapour annealing. 

Recent studies report potential improvement of solar cell performance by shifting towards mixed dimensional structures (0D/2D) [2,36]. The characteristics of 0D, 2D and (0D/2D) mixed dimensional structures through molar ratio variation were thoroughly investigated by Johansson et al. [36]. Good optoelectronic properties were observed at the ratio CsI:${\mathrm{BiI}}_{3}\;\mathrm{of}\;1:1\;$and 1:2. 0D hexagonal structure at ratio 1.5:1 was characterised with a large indirect bandgap (2.07 eV) and the lowest power conversion efficiency (0.07%). Addition of CsI to form 1:1, 1:2 and 1:3 ratios created a mixed 0D/2D structure. The structural transformation is mainly due to reallocation of the adjacent BiI_6_ octahedral from the face-sharing position to corner sharing along a and b directions. The Fermi levels are also shifted closer to the valence band due to the high amount of metallic Bi-inducing p-type doping material. The power conversion efficiency with mixed dimensionality was found to be higher than that of the pure structures. The best-performing cell was obtained at 1:1 ratio with PCE of 0.62%. The bandgap decreases from 2.07 eV with 1.5:1 molar ratio to 1.77 eV for the rest of the dimensionality structures.

In this study, caesium bismuth iodide solar cell was fabricated at the new ratio of 1:1.5 or simply 2:3 to create a mixed dimensional structure by the solution method. This ratio is the inverse of ratio 1.5:1 for the commonly known and well-studied ${\mathrm{Cs}}_{3}{\mathrm{Bi}}_{2}I_{9}$. The solvent vapour annealing method was further employed to enhance crystallinity and surface morphology using a polar organic solvent mixture of $\mathrm{DMF}/{\mathrm{CH}}_{3}\mathrm{OH}\;$vapour at a volume ratio of 1/4. The optical, structure and electrical properties were investigated. The cell with the best performance had a power conversion efficiency of 0.98%. The cell structure was further characterised by a higher photocurrent and extended visible light absorption spectrum at a longer wavelength. The cell covered a visible spectrum greater than 720 nm, which exceeded that of ${\mathrm{Cs}}_{3}{\mathrm{Bi}}_{2}I_{9}$ and$\;{\mathrm{CsBi}}_{3}I_{10}$. The solar cell device maintained 86% of its efficiency without encapsulation for 20 days. This research opens a new avenue for investigating the potentiality of lead-free bismuth-based perovskites for solar cell applications and other optoelectronic uses.

## 2. Materials and Methods

### 2.1. Chemicals and Reagents

CsI (99.9%), acetonitrile (99.5%) and chlorobenzene (99.8%, anhydrous) were obtained from Acros, while bismuth (III) iodide (99%), dimethyl sulphoxide (DMSO, anhydrous, ≥99.9%), acetylacetone (99.6%), Bis (trifluoromethane) sulphonamide lithium salt (99.95%, Li-TFSI, trace metals basis), Spiro-OMeTAD (99% HPLC) and methanol (99.9%) were purchased from Sigma Aldrich. *N*-*N* dimethylformamide (DMF, anhydrous, 99.8%) was obtained from Alfa Aser,4-tert-butyl pyridine was purchased from Aladdin, FK 209-cobalt(III)-TFSI was purchased from MaterWinChemicals and tris(2-(1H-pyrazol-1-yl)-4-tert-butylpyridine)cobalt(III)tris(trifluoromethylsulfonyl)imide) was obtained from Aladdin while isopropanol was from J&K Scientific and Dyesol 30 NR-D (Queanbeyan, Australia). All chemicals were utilised directly without further purification.

### 2.2. Solar Cell Device Fabrication

**Figure 1:**
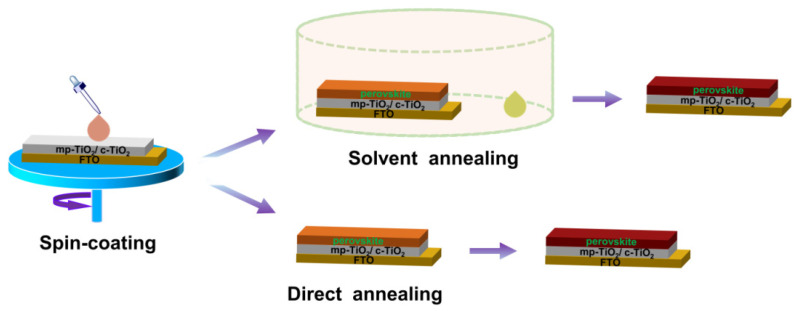
Method employed for the fabrication of caesium bismuth iodide solar cell.

A device structure glass/FTO/c-TiO_2_/m-TiO_2_/perovskite/Spiro-OMeTAD/Au was fabricated with or without solvent vapour annealing, as shown in Figure 1. In the ultrasonic cleaner (Hwotech, BZS250GF-TS, 250 W power), fluorine-doped tin oxide (FTO) glass substrates were sequentially cleaned successively with deionised water and ethanol prior to UV/O_3_ treatment for 20 min on each step. Then, a compact TiO_2_ layer was deposited on a clean substrate by spray pyrolysis using 0.6 mL of titanium diisopropoxide bis(acetylacetone) dissolved in 7 mL isopropanol and 0.4 mL acetylacetone. Dyesol 30 NR-D was then dissolved in ethanol solution (1/3.5), (*w*/*w*) and then spin-coated at 4000 rpm for 30 s followed by sintering at 460 °C for 1 h on a hot plate to form the mesoporous TiO_2_ layer. 

Perovskite precursor solutions were prepared by mixing CsI and BiI_3_ at a molar ratio of 1:1.5 and dissolving in DMF alone or the mixture of DMF: DMSO (9:1) (*v*/*v*) to form 1M solution. The solutions were magnetically stirred at 70 °C for 18 h with DMF only and for 30 min with a mixture of DMF and DMSO. Caesium bismuth iodide was further prepared at a molar ratio of CsI/BiI_3_, 1.5:1 and 1:3 in DMF solution to form 1M solution for comparison purposes. The perovskite precursor solution was spin-coated on top of the electron transport layer (FTO/c-TiO_2_/m-TiO_2_) at 3000 rpm for 30 s followed by annealing with and without DMF/CH_3_OH vapour (1:4) (*v*/*v*) at various annealing temperatures (125, 140, 160 °C) for 30 min.

The hole transport layer was formed by spin-coating Spiro-OMeTAD onto the perovskite layer at 4000 rpm for 20 s. The Spiro-OMeTADprecursor solution was prepared by dissolving 72.3 mg of Spiro-OMeTAD mixed with 28.8 µL of tert-butylpyridine, 17.5 µL of a Li-TFSI solution formed from 520 mg mL^−1^ bis(trifluoromethane)sulphonamide lithium salt dissolved in acetonitrileand 8 µL of an FK209-cobalt(III)-TFSI solution in 300 mg mL^−1^ FK209-cobalt(III)-TFSIin acetonitrile in 1 mL ofchlorobenzene. The thermal evaporation method was used to deposit a 70 nm thick gold electrode at a pressure of 4 × 10^−4^ Pa. Then, a black mask defined a 1 mm^2^ (1.66 × 0.60 cm^2^) active layer for characterisation.

### 2.3. Characterisation

The J-V characteristics were obtained using a Keithley 2400 source meter aligned with a sunlight simulator (XES-300T1, SAN-EI Electric, AM 1.5G, Kamishinjo, Higashiyodogawa-ku, Osaka, Japan). Standard silicon solar cell was used for calibration. Surface film morphology (SEM images) was obtained using an SU8010SEM (Hitachi, Matsuda, Japan). XRD patterns were recorded using an X-ray diffractometer (XRD, SmartLab, Rigaku Corp., Tokyo, Japan) with Cu-Kα radiation (1.5418). All characterisation was carried out in ambient air without encapsulation. Afield emission scanning electron microscope (FE-SEM, sirion200, FEI Corp., Eindhoven, Holland) was used to examine the film’s surface morphology, while a Shimadzu UV-2450 spectrophotometer (Shimadzu Corp., Kyoto, Japan) was used to record the films’ UV-vis spectra.

## 3. Results

### 3.1. SEM Analysis

**Figure 2:**
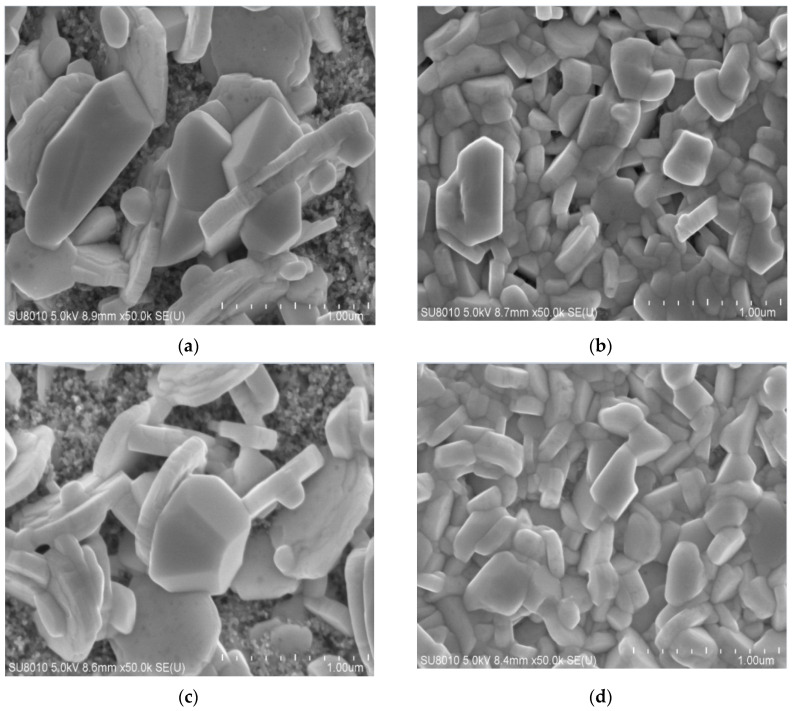
SEM images for the films annealed: (**a**) with DMF only; (**b**) with the mixture of DMF and DMSO; (**c**) with DMF only under DMF/CH_3_0H solvent vapour annealing; and (**d**) with DMF and DMSO under DMF/CH_3_0H solvent annealing.

SEM images for solar cells fabricated with DMF alone show rough morphology with large crystals grains and hexagonal flakes growing parallel to the surface. The scaffold layer of $m-{\mathrm{TiO}}_{2}$ is clearly visible in Figure 2a, indicating that the perovskite layer on the substrate is not completely covered. Similar findings have been reported by previous researchers [28,36,37,38]. In the DMF solution, perovskite precursors form an intermediate complex, which induces dissolution—recrystallisation based on Ostwald ripening theory in which small grains are redissolved and redeposited on large grains [39]. When DMF is used alone without anti-solvent treatment, the rate of grain growth exceeds the rate of nucleation, resulting in the island growth of film with large grains. Furthermore, no significant improvement was observed when DMF was used in parallel with DMF/CH_3_OH vapour annealing as shown in Figure 2b. The combination of DMF with DMSO tends to reduce the grain size and, as a result, increases film packing density, resulting in the formation of a more compact film. Figure 2c shows that the cells made by mixed solvent have more uniform grains and smaller grains with higher packing density than cells made with DMF alone. Pinholes, on the other hand, can be seen. Results of improving film morphology using the mixed solvents (DMF: DMSO) on bismuth-based perovskite films at the required proportion have been reported elsewhere [34]. As a coordinating molecule, DMSO reduces the fast interaction between CsI and BiI_3_ by donating a lone-pair electron on oxygen to form an intermediate complex which produces perovskite film with compact grains and small size upon annealing [39,40,41].

Cells fabricated with the mixture of DMF and DMSO, followed by solvent annealing under $\mathrm{DMF}:{\mathrm{CH}}_{3}\mathrm{OH}$ vapour, demonstrated good film morphology, as shown in Figure 2d. DMF dissolves the formed perovskite and the low boiling point of ${\mathrm{CH}}_{3}\mathrm{OH}$ allows quick supersaturation and precipitation, enabling fast nucleation and rapid crystal growth, resulting in uniform film with good reproducibility. The DMF/CH_3_OH vapour increases the packing density, resulting in improved film morphology. Solvent annealing with solvent vapour rather than solvent dripping while spinning for lead-free perovskites has also been reported to improve the quality of film morphology elsewhere [33,35,39].

### 3.2. The Influence of Solvent Vapour Annealing on I–V Characteristics

**Figure 3:**
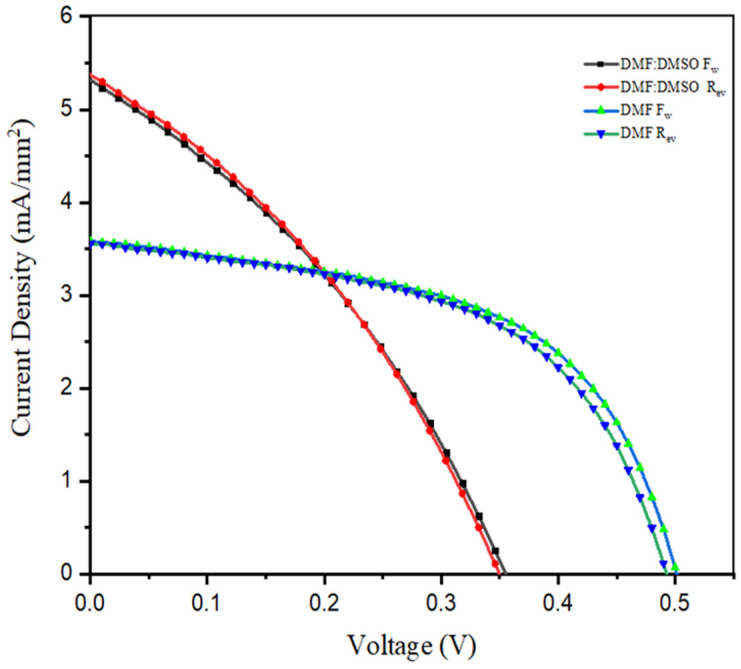
The influence of precursor solvent in open-circuit voltage and current density.

**Figure 4:**
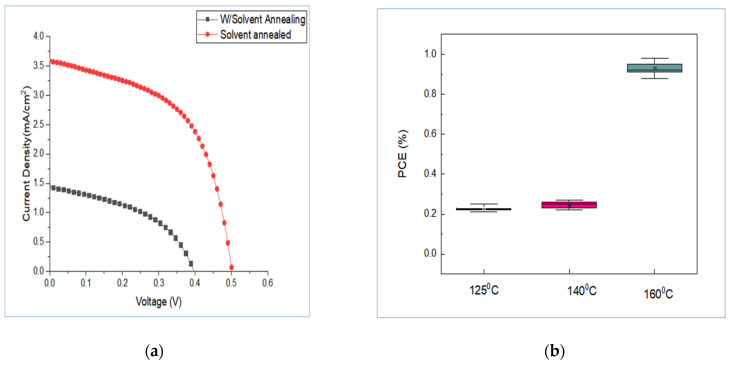
(**a**) I–V characteristics for the cells annealed with and without solvent vapour annealing at 160 °C, while (**b**) shows the effect of annealing temperature on PCE under solvent vapour annealing for the solar cells fabricated under DMF solution.

**Figure 5:**
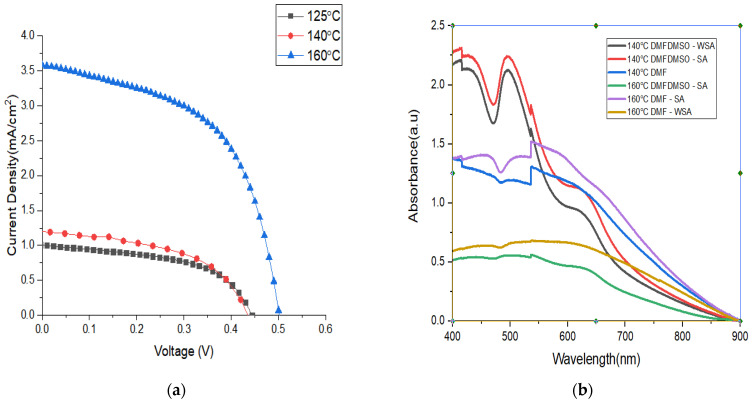
(**a**) The effect of annealing temperature on I–V characteristics; (**b**) effect of solvent type on absorption spectrum.

The I–V characteristics for the device structure FTO/c-TiO_2_/mp-TiO_2_/caesium bismuth iodide/Spiro-OMeTAD/Au demonstrate that the mixture of DMF and DMSO improves the current density while decreasing the open-circuit voltage. However, as shown in Figure 3, the cell fabricated solely with DMF had a higher open-circuit voltage but a lower current density. When comparing cells made with and without solvent vapour annealing, the cells made with DMF/CH_3_OH vapour have higher open-circuit voltage and current density, as shown in Figure 4a and Table 1. Solvent vapour annealing tends to enlarge the grain size, allowing the charges to be transported and collected through a single grain without encountering grain boundaries. It further increases the charge recombination lifetime by reducing trap density, resulting in increased charge carrier mobility and faster charge extraction [42]. Yu et al. [43] reported that solvent vapour annealing on lead-based perovskite solar cells causes a red shift in absorption spectrum and extended absorption capacity to 800nm. This is due to the fact that crystallinity of the perovskite film is an important parameter that is related to the perovskite’s light absorption, charge transport and recombination characteristics. High crystallinity means that the resulting devices will perform well [43]. The influence of solvent type has been found to affect solar cell absorption capacity, as seen in Figure 5b. Solar cells which have been thermally annealed under solvent vapour annealing have higher absorption capacity with extended absorption wavelength than the cells annealed without solvent vapour annealing.

The annealing temperature was also found to affect solar cell performance, as shown in Figure 4b and Figure 5a. The performance of the cell fabricated with DMF only at 160  ℃ was superior to that of the cells fabricated at 140  ℃ and 125  ℃. Therefore, annealing temperature of 160 ℃ was adopted for fabrication of the rest of the solar cells for characterisation. With the mixture of DMF and DMSO, the highest efficiency was obtained when cells were annealed at a temperature of 160 ℃.

### 3.3. The UV-Vis Measurements

**Figure 6:**
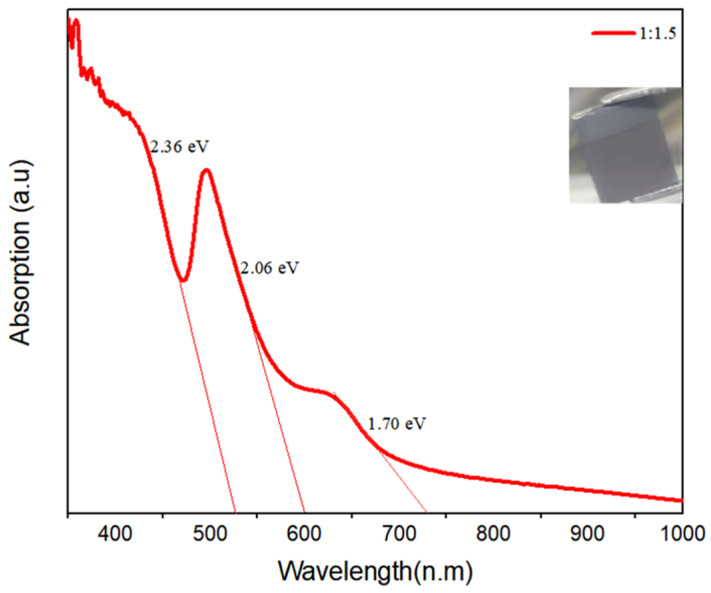
Absorption spectra for the caesium bismuth iodide fabricated at CsI/BiI_3_ ratio of1/1.5.

To analyse the corresponding absorption capacity, UV-Vis measurements were performed on the absorber material fabricated at a new ratio of CsI/BiI_3_ (1/1.5). The solar absorber material had a higher photocurrent and more extensive visible light absorption spectrum at a longer wavelength. Higher photocurrent and extended visible light absorption spectrum at longer wavelength characterised the improved solar absorber structures. As shown in Figure 6, the solar cell absorber material covered a visible spectrum greater than 730 nm, outperforming Cs_3_Bi_2_I_9_ and CsBi_3_I_10_. The new ratio produced a blackish film with a bandgap of 1.70 eV and additional excitation absorption peaks at 2.06 eV and 2.36 eV. Previous researchers have also reported the formation of additional excitation absorption peaks on caesium-bismuth-iodide-based solar cells [29,33].

The relatively modest power conversion efficiency reported in this study might be contributed by the nature of the hole transport material used and the quality of film morphology. Spiro-OMeTAD is used in this study due to its high solubility, appropriate oxidation potential, broad absorption spectrum and amorphous structure [44]. However, pristine wide bandgap Spiro-OMeTAD has low conductivity and low mobility. As a result, P-type dopants such as lithium salt, 4-tert-butylpyridine (TBP) and cobalt complexes are commonly added to improve conductivity and reduce charge recombination [34,44]. These additives add challenges to the performance of hole-transporting materials by inducing degradation on charge-selective layers. The hygroscopic nature of dopants makes the hole-transporting layer highly hydrophilic, causing chemical degradation and compromising the device’s stability [45,46,47]. Johansson et al. [36] reported that 4-tert-butyl pyridine in Spiro-OMeTAD tends to dissolve the CsBi_3_I_10_ perovskite layer and hence is not suitable for 0D/2D lead-free bismuth-based perovskite materials. P3HT on the other hand, leads to low open circuit voltage due to its disadvantageous energy level. Previous researchers recommended the use of dopant-free or inorganic hole transport materials for bismuth-based compounds [32,33]. Most of the cells fabricated by using inorganic hole transport material such as CuI and NiOx displayed power conversion efficiency greater than 1% as seen in Table 2. Similarly, as reported for lead-based perovskites, non-wetting hole transport layers have great potential in suppressing heterogeneous nucleation and facilitating crystal growth with large grains and with less drag force, which dramatically reduces charge recombination [48,49]. The electronic properties and power conversion efficiency of both organic and inorganic electronic materials are heavily influenced by material crystallinity. Through addition to thermal annealing, solvent annealing, which involves the introduction of solvent vapour during the crystallisation of bulk or thin film materials, has been found to be an effective method for increasing the crystallinity of some very specific organic semiconductors.

### 3.4. XRD Characterisation

**Figure 7:**
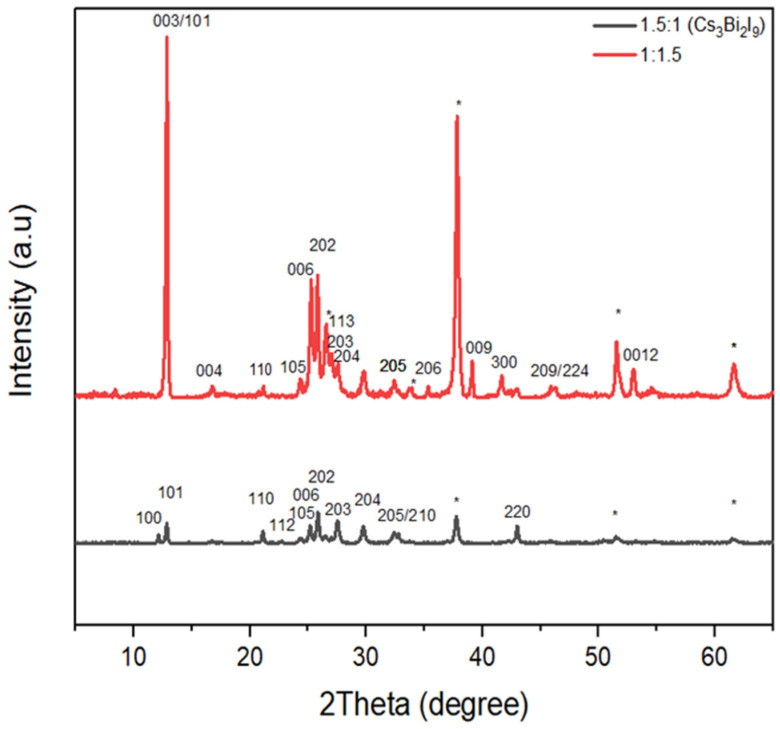
XRD comparisons for caesium bismuth iodide perovskite formed at (CsI/BiI_3_) ratios 1.5:1 and 1:1:5. * Caesium bismuth iodide at ratio 1:1.5, on the other hand, was found to have additional characteristics peaks at 009,012,209 and 300, indicating the growth of another phase.

**Figure 8:**
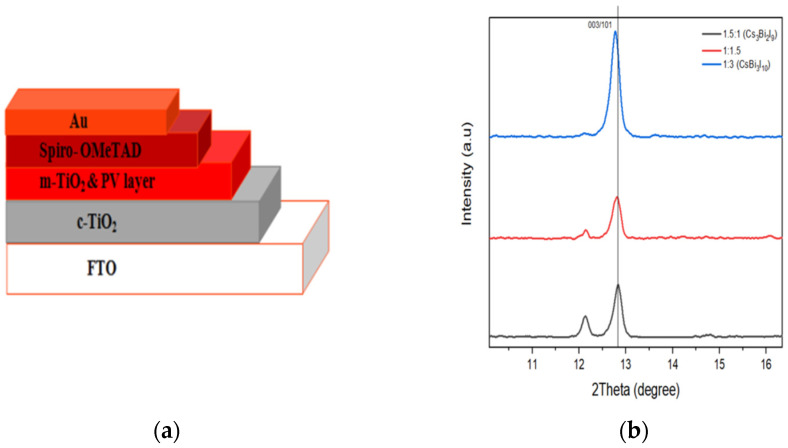
(**a**) Solar cell configuration; (**b**) XRD comparison for molar ratio 1:1.5, 1.5:1 and 1:3.

Films were made with different molar ratios of CsI/BiI_3_ for crystal comparison, with ratios of 1.5:1, 1:1.5 and 1:3. The normal ${\mathrm{Cs}}_{3}{\mathrm{Bi}}_{2}I_{9}\;$at a ratio 1.5:1 was found to adhere to a 0-dimensional structure with characteristic peaks at 100, 101, 110, 105, 202, 203, 204, 205 and 220 and space group P63/mmc and lattice parameters a = 8.409 Å and c = 21.243 Å (Figure 7). These findings corroborate the previous ones [36,37]. Caesium bismuth iodide at ratio 1:1.5, on the other hand, was found to have additional characteristics peaks at 009, 012, 209 and 300, indicating the growth of another phase. According to Johansson et al. [36], any sample fabricated using intermediate precursor ratios (1:1, 1:2, 1:3 and 1:9) contains a heterogeneous mixed phase with 0D/2D structure. As a result, this new compound also has a 0D/2D structure. Figure 8b shows that at 2θ=12.8°, the film with CsI/BiI_3_ at ratio 1:3 is right-skewed, whereas at ratios of1:1.5 and 1.5:1, it orients almost at the same plane, indicating that it resembles Cs_3_Bi_2_I_9_ more than CsBi_3_I_10_. 

### 3.5. Stability Analysis

**Figure 9:**
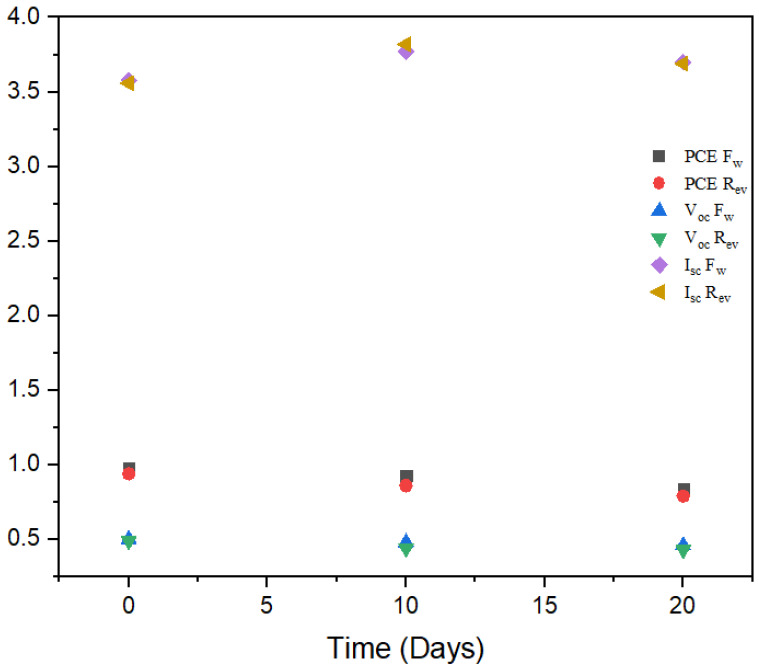
I_sc_, V_oc_, FF and PCE of perovskite solar cell stored for 20 days.

Figure 9 shows that after 20 days, the best solar cell device fabricated by new ratio 1:1.5 stored in a nitrogen glove box maintained 86 percent of its efficiency without encapsulation, with only 5 percent decay occurring within the first ten days. The previous researcher reported similar results for the stability of 0/2-dimensional structures [35]. The V_oc_ was almost constant, while the current density increased slightly with age. 

## 4. Conclusions

In conclusion, we fabricated a 0D/2D mixed dimensional caesium bismuth iodide solar cell with CsI/BiI_3_ at a molar ratio of 1:1.5. This intermediate precursor ratio of 1:1.5 produced a heterogeneous mixed phase with 0D ${\mathrm{Cs}}_{3}{\mathrm{Bi}}_{2}I_{9}$ and 2D ${\mathrm{BiI}}_{3}$. The cell was further discovered to have an extended absorption capacity of more than 720 nm. According to SEM images, DMF/CH_3_OH solvent vapour annealing improves crystallinity and surface morphology. The power conversion efficiency was found to increase from 0.26% to 0.98% upon solvent vapour annealing. The cell samples fabricated with the mixture of DMF and DMSO display higher current density than samples fabricated with DMF only. The XRD pattern further revealed that samples fabricated at a ratio of 1:1.5 had greater resemblance with 1.5:1 than with 1:3. The results are promising when compared to other mixed 0D/2D cell structures. The use of Spiro-OMeTAD as the hole transport material, on the other hand, maybe a hindrance to the limited efficiency.

This research will pave the way for developing stable, non-toxic and hysteresis-free, lead-free, all-inorganic perovskite solar cells.

## Figures and Tables

**Table 1:** Influence of annealing condition and solvent type on I–V characteristics.

Solvent Type	Annealing Condition	Scan.	$\;V_{oc}$ (V)	$\;J_{sc}$ $\;mA/cm^{2}\;$	FF (%)	PCE (%)
DMF only At 160 °C	CA	Reverse	0.37	1.42	46.13	0.25
		Forward	0.40	1.44	45.08	0.26
	with SVA	Reverse	0.49	3.56	53.53	0.94
		Forward	0.50	3.58	54.42	0.98
DMF:DMSO (9:1) At 140 °C	CA	Reverse	0.35	1.41	32.11	0.16
		Forward	0.35	1.38	31.14	0.15
	with SVA	Reverse	0.35	5.37	34.60	0.65
		Forward	0.36	5.32	34.10	0.65

**Table 2:** Photovoltaic performance of lead-free bismuth-based perovskite solar cells so far fabricated.

Compound	Device Structure	Jsc (mAcm^−2^)	Voc (V)	FF%	PCE (%)	REF
Cs_3_Bi_2_I_9_	FTO/c-TiO_2_/m-TiO_2_/Cs_3_Bi_2_I_9_/Spiro-OMeTAD/Ag	2.15	0.85	60	1.09	[28]
Cs_3_Bi_2_I_9_	FTO/c-TiO_2_/m-TiO_2_/Cs_3_Bi_2_I_9_/P3HT/Ag	0.18	0.26	37	0.02	[29]
CsBi_3_I_10_	FTO/c-TiO_2_/m-TiO_2_/CsBi_3_I_10_/P3HT/Ag	3.40	0.31	38	0.4	[29]
Cs_3_Bi_2_I_9_	FTO/c-TiO_2_/m-TiO2/Cs_3_Bi_2_I_9_/Spiro-OMeTAD/Au	0.67	0.49	64	0.21	[30]
CsBi_3_I_10_	FTO/c-TiO_2_/m-TiO_2_/CsBi_3_I_10_/P3HT/Au	2.40	0.34	44	0.36	[31]
CsBi_3_I_10_	FTO/c-TiO_2_/m-TiO_2_/CsBi_3_I_10_/P3T1/Au	2.60	0.47	38	0.47	[31]
CsBi_3_I_10_	FTO/c-TiO_2_/m-TiO_2_/CsBi_3_I_10_/TQ1/Au	2.38	0.62	52	0.77	[31]
Cs_3_Bi_2_I_9_	FTO/c-TiO_2_/Cs_3_Bi_2_I_9_/Spiro-OMeTAD/Au	4.45	0.79	50	1.77	[32]
Cs_3_Bi_2_I_9_	FTO/c-TiO_2_/Cs_3_Bi_2_I_9_/PTAA/Au	4.82	0.83	57	2.3	[32]
Cs_3_Bi_2_I_9_	FTO/c-TiO_2_/Cs_3_Bi_2_I_9_/CuI/Au	5.78	0.86	64	3.2	[32]
Cs_3_Bi_2_I_9_	FTO/c-TiO_2_/m-TiO_2_/m-ZrO_2_/Cs_3_Bi_2_I_9_/C	4.75	0.46	69	1.51	[50]
Cs_3_Bi_2_I_9_	ITO/NiOx/Cs_3_Bi_2_I_9_/PCBM/C60/BCB/Ag	0.51	0.75	59	0.23	[51]
Cs_3_Bi_2_I_6_Br_3_	ITO/NiO_x_/Cs_3_Bi_2_I_6_Br_3_/PCBM/C60/BCB/Ag	3.15	0.64	57	1.15	[51]
Cs_3_Bi_2_I_9_	ITO/PTAA/Cs_3_Bi_2_I_9_/PCBM/AZO/Ag	1.76	0.47	45	0.37	[33]
Cs_3_Bi_2_I_9_	ITO/PEDOT:PSS/Cs_3_Bi_2_I_9_/PCBM/AZO/Ag	0.54	0.38	35	0.073	[33]
Cs_3_Bi_2_I_9_	ITO/NiOx/Cs_3_Bi_2_I_9_/PCBM/AZO/Ag	3.42	0.74	51	1.26	[33]
CsBi_3_I_10_	FTO/c-TiO_2_/m-TiO2/CsBi_3_I_10_/Spiro-OMeTAD/Ag	4.45	0.55	42	1.03	[35]
Cs_3_Bi_2_I_9_	AZO/c-TiO_2_/Cs_3_Bi_2_I_9_/CuSCN/graphite	1.43	0.37	32	0.17	[52]
Cs_a_Bi_b_I_x_	FTO/c-TiO_2_/m-TiO_2_/Cs_a_Bi_b_I_x(1:1)_/TQ1/Au	2.22	0.57	49	0.62	[36]
Cs_a_Bi_b_I_X_	FTO/c-TiO_2_/m-TiO_2_/Cs_a_Bi_b_I_x(1:2)_/TQ1/Au	2.79	0.43	42	0.5	[36]
Cs_a_Bi_b_I_X_	FTO/c-TiO_2_/m-TiO_2_/Cs_a_Bi_b_I_x(1:3)_/TQ1/Au	3.18	0.37	40	0.47	[36]
Cs_a_Bi_b_I_X_	FTO/c-TiO_2_/m-TiO_2_/Cs_a_Bi_b_I_x(1.5:1)_/TQ1/Au	0.29	0.68	33	0.07	[36]
Cs_a_Bi_b_I_X_	FTO/c-TiO_2_/m-TiO_2_/Cs_a_Bi_b_I_x(1:1.5)_/Spiro-OMeTAD/Au	3.58	0.50	54	0.98	This study

## Data Availability

The data presented in this study are available on request from the corresponding author.
